# Finite element model of mechanical imaging of the breast

**DOI:** 10.1117/1.JMI.9.3.033502

**Published:** 2022-05-23

**Authors:** Rebecca Axelsson, Hanna Tomic, Sophia Zackrisson, Anders Tingberg, Hanna Isaksson, Predrag R. Bakic, Magnus Dustler

**Affiliations:** a Lund University, Skåne University Hospital, Medical Radiation Physics, Department of Translational Medicine, Malmö, Sweden; b Lund University, Skåne University Hospital, Diagnostic Radiology, Department of Translational Medicine, Department in Imaging and Functional Medicine, Malmö, Sweden; c Lund University, Department of Biomedical Engineering, Lund, Sweden; d University of Pennsylvania, Department of Radiology, Philadelphia, Pennsylvania, United States

**Keywords:** breast cancer, mammography, finite element, mechanical imaging, virtual clinical trial

## Abstract

**Purpose:**

Malignant breast lesions can be distinguished from benign lesions by their mechanical properties. This has been utilized for mechanical imaging in which the stress distribution over the breast is measured. Mechanical imaging has shown the ability to identify benign or normal cases and to reduce the number of false positives from mammography screening. Our aim was to develop a model of mechanical imaging acquisition for simulation purposes. To that end, we simulated mammographic compression of a computer model of breast anatomy and lesions.

**Approach:**

The breast compression was modeled using the finite element method. Two finite element breast models of different sizes were used and solved using linear elastic material properties in open-source virtual clinical trial (VCT) software. A spherical lesion (15 mm in diameter) was inserted into the breasts, and both the location and stiffness of the lesion were varied extensively. The average stress over the breast and the average stress at the lesion location, as well as the relative mean pressure over lesion area (RMPA), were calculated.

**Results:**

The average stress varied 6.2–6.5 kPa over the breast surface and 7.8–11.4 kPa over the lesion, for different lesion locations and stiffnesses. These stresses correspond to an RMPA of 0.80 to 1.46. The average stress was 20% to 50% higher at the lesion location compared with the average stress over the entire breast surface.

**Conclusions:**

The average stress over the breast and the lesion location corresponded well to clinical measurements. The proposed model can be used in VCTs for evaluation and optimization of mechanical imaging screening strategies.

## 1 Introduction

Breast cancer is the most common cause of cancer death in women worldwide, and roughly 2.3 million women are diagnosed every year.^1^ In many countries, breast cancer screening with digital mammography (DM) has been implemented for early detection, with the goal of diminishing the risk of death from breast cancer.^2^^,^^3^ Despite a reduction in breast cancer mortality, DM has known limitations related to the number of false positive (FP) and false negative (FN) results.^4^

New techniques dealing with the limitations of DM are being evaluated, with one of the most promising being digital breast tomosynthesis (DBT). DBT is pseudo 3D imaging of the breast and has been shown to increase cancer detection in comparison with DM.^4^ This is because DBT images offer decreased anatomical noise caused by overlapping anatomical structures. Moreover, several studies have shown that DBT performs better than DM in a screening setting,^4^^–^^7^ but with varying effects on the FP rate. The potential increase in FP is of importance, for example, in Europe, where the FP rate is relatively low. In addition, it takes more time for radiologists to read a stack of reconstructed DBT slices than DM images.^7^

A new emerging imaging technique is mechanical imaging (MI), which is the focus of this study. MI is a technique that measures the stress distribution over the compressed breast during mammography. MI has shown the ability to distinguish between malignant and benign findings, based on differences in the tissue’s mechanical properties.^8^ Benign lesions and normal tissue are generally softer than malignant lesions. Malignant lesions can be up to 30 times stiffer than adipose tissue^9^ and roughly four times stiffer than benign lesions.^10^

A study by Dustler et al.^11^ showed that MI could potentially reduce the number of recalled women by 36% and the number of biopsies by 32% if introduced into clinical practice. The potential reduction in recall rates could alleviate the unnecessary stress and psychological discomfort experienced by women recalled from breast cancer screening for further investigations.^12^ Moreover, MI in combination with DM and DBT has the potential for increasing cancer detection while reducing the number of false positives.

Evaluation and optimization of the MI-based screening techniques can be performed using virtual clinical trials (VCTs). VCTs are preclinical tools in the form of computer-simulated clinical trials. VCTs simulate anatomy, imaging techniques, and image interpretation, and this makes them both a cost and time-effective tool for preclinical evaluation and optimization of medical imaging systems.^13^

VCTs for breast cancer screening require simulated models of breast tissue. Many studies have used finite elements (FE) to model breast compression, using different medical imaging modalities to construct the anatomy of the breast. Several studies used magnetic resonance (MR) imaging data of the breast to create the FE model of the breast anatomy.^14^^,^^15^ Breasts containing lesions have also been modeled, using both linear and hyperelastic material models to model breast tissue^16^ (and were validated by comparing the contour of mammograms with the corresponding deformed contour of the FE model).^17^ Others have used computed tomography (CT) images to develop the FE model of the breast.^18^ Also using CT to model the breast anatomy, Kellner et al.^19^ used a linear elastic material model with the breast consisting of adipose tissue, glandular tissue, and skin. The Elastic modulus was 1 kPa for adipose tissue, 10 kPa for glandular, and 88 kPa for skin, respectively, based on literature data.^20^^–^^23^ Breast tissue has been modeled as linear elastic by other researchers as well^24^ and more recently in the Virtual Imaging Clinical Trial for Regulatory Evaluation (VICTRE).^25^ Moreover, the breast can be modeled using virtual phantoms based on breast anatomy,^25^ which has been done in this study. Its advantages are the ability to customize and modify breast anatomy for a variety of different tissue compositions.

The aim of this study is to simulate the acquisition of MI by modeling breast compression using the finite element method. We validate the results against the average stress values from available clinical MI data. Our model of MI acquisition could be used for future preclinical optimization of MI systems.

## 2 Clinical Mechanical Imaging Acquisition

**Fig. 1:**
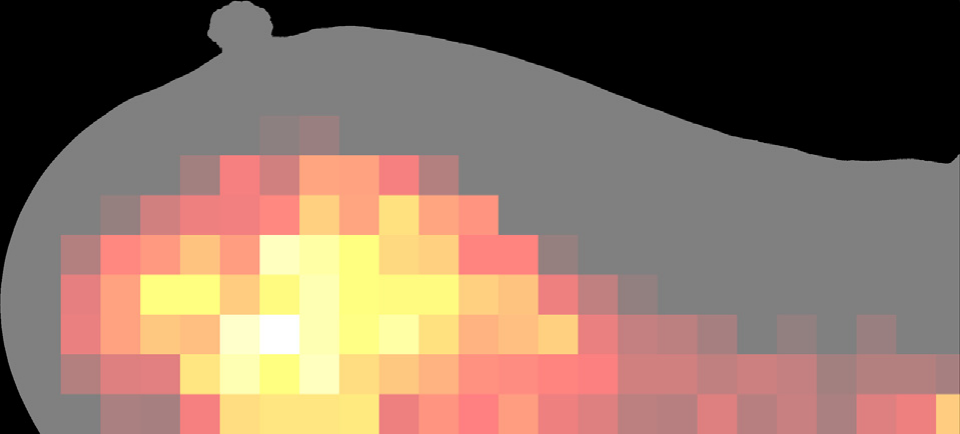
Stress map of a breast as seen clinically, in MLO view (this stress map was selected from a clinical study by Dustler et al.^27^). The darker areas correspond to areas of low stress, and the bright areas correspond to areas of high stress.

When MI is acquired in clinical investigations, the stress over the breast is recorded using a thin, flexible sensor (Tekscan, Boston, Massachusetts, United States).^26^ The sensor records the stress in the direction of compression (see Fig. 1). MI is done with simultaneous x-ray imaging of the breast, either with digital mammography or digital breast tomosynthesis.

In a study by Dustler et al.,^11^ the relative mean pressure over lesion area (RMPA) was used as a metric to differentiate benign from malignant lesions. The RMPA was defined as the average stress of 3×3 sensor elements (∼1  cm×1  cm each) centered over the lesion, relative to the average stress overall sensor elements that fully covered the breast (i.e., stress over the background).

MI depends on multiple factors, both anatomical and technological. The effect of individual factors cannot be assessed using clinical data only, due to different factors, e.g., breast size, volumetric breast density, etc. This motivates our simulation approach.

## 3 Material and Methods

### 3.1 Breast Anatomy Simulation

**Fig. 2:**
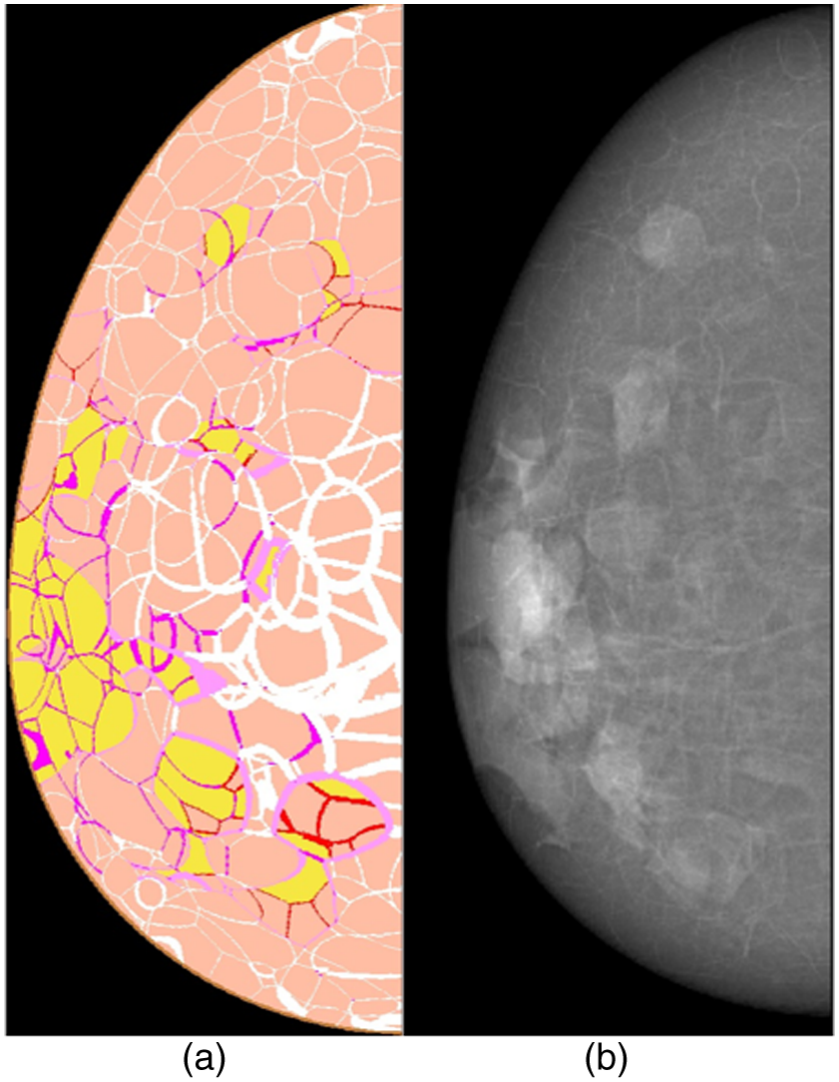
An example of a phantom from OpenVCT: (a) the phantom interior and (b) the corresponding 2D x-ray projection of the phantom.

An FE model based on the breast anatomy from OpenVCT (developed at the University of Pennsylvania, Philadelphia, Pennsylvania, United States)^28^^,^^29^ was implemented using an open-source FE solver (FEBio,^30^ University of Utah, Salt Lake City, Utah, United States). This simulated the stress and strain distribution on the breast surface with the purpose of simulating MI acquisition. The breast phantoms are voxel-based and mimic real breast anatomy, consisting of adipose tissue, fibroglandular tissue, Cooper’s ligaments, and skin (see Fig. 2).

The breast outline is formed by two ellipsoid quarters joined at the nipple plane, with a planar approximation of the chest wall. The breast models used in this study were of two different volumes, roughly corresponding to bra cups A and B.^31^ The model of cup A had dimensions of 170×75×37  mm (coronal, transversal, and sagittal plane) and a volume of ca 250 mL [Fig. 4(a)]. The model of cup B had dimensions of 170×100×50  mm (coronal, transversal, and sagittal plane), and a volume of ca 450 mL [Fig. 4(b)]. The meshing of the models was performed with the open-source iso2mesh software,^32^ based on works by Garcia et al.^33^ and Fedon et al.^34^ For meshing, the virtual phantoms (cups A and B) were sliced to create a stack of images, representing the volume of the breast phantom, which were then used as input in the iso2mesh software to create a mesh. The cup A model consisted of 1,72,541 tetrahedral elements and 38,360 nodes, and the cup B model consisted of 2,93,914 tetrahedral elements and 61,570 nodes.

### 3.2 Mechanical Properties of the Tissue and Lesion

The mechanical properties of the simulated breast tissue were taken from OpenVCT.^35^ In OpenVCT, mammographic compression is modeled by assuming uniform mechanical properties throughout the breast. In our study, the tissue was assumed to be linear elastic with an Elastic modulus of 12.75 kPa and a Poisson’s ratio of 0.49.^36^ The parameters chosen are within the range of values reported for adipose tissue and similar to what others have assumed for FE based work.^10^^,^^35^

Simulated lesions were inserted in the computer breast models by selecting a spherical volume of interest in the breast mesh. The elements within the volume of interest were assigned the desired lesion properties. The lesion stiffness was assigned as 15, 30, or 50 times higher than the surrounding adipose tissue, based on previously reported values.^10^^,^^37^ The lesion diameter was assumed to be 15 mm, which is within the reported range of sizes of intraductal carcinoma.^38^

### 3.3 Varying Lesion Location

**Fig. 3:**
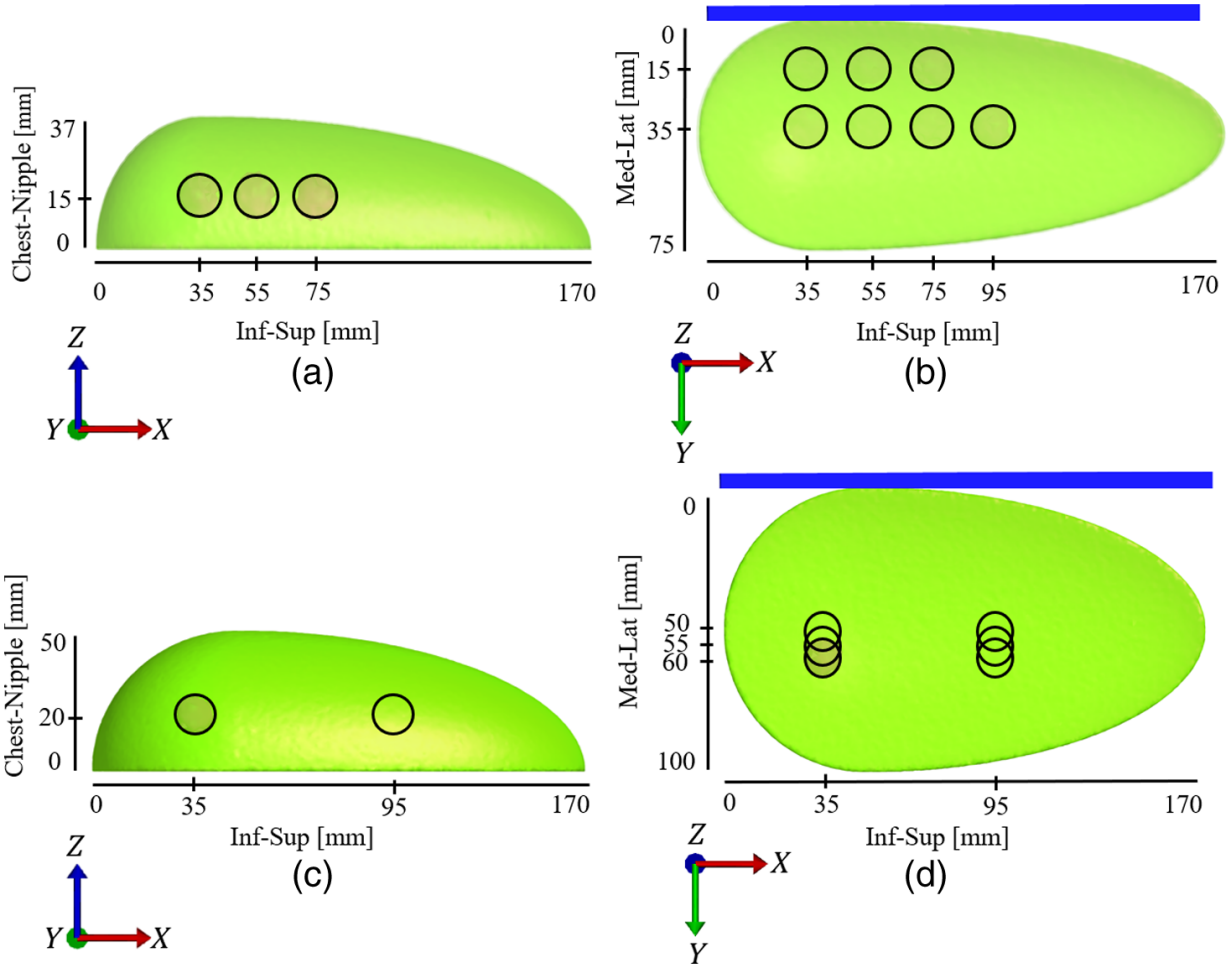
Breast anatomies and dimensions for cup A (a, b) and cup B (c, d). The blue plate marks the compression plate. In (a, b) the circles mark the lesion locations. In (c, d) the circles mark the start and endpoints of the interval of lesion locations investigated. Within this interval, the lesion locations are in increments of 5 mm. (a) Lesion locations in the cup A breast in the inferior-superior direction and chest-nipple direction (coronal-sagittal plane). (b) Lesion locations in the cup A breast in the inferior-superior direction and medial-lateral direction (coronal-transversal plane). (c) Lesion locations in the cup B breast in the inferior-superior direction and chest-nipple direction (coronal-sagittal plane). (d) Lesion locations in the cup B breast in the inferior-superior direction and medial-lateral direction (coronal-transversal plane).

For the cup A model, the lesion location was varied in the x- (inferior-superior direction) and y- (medial-lateral direction) directions in increments of 20 mm [Figs. 3(a) and 3(b)]. All lesions were contained within the breast and with a margin of at least 7.5 mm to the breast surface. Thus, the lesion location could not be varied in the z-direction (chest-nipple direction).

The lesion location for cup B corresponded to the locations in the x- and y-directions in cup A and varied in increments of 5 mm [Figs. 3(c) and 3(d)]. The lesion location was also varied in the z-direction, being 5 mm closer to the chest wall (z-direction) and moved in 5 mm increments in the x-direction and in the middle of the breast [50 mm from the compression plate in Fig. 3(b)]. Lesions were located a minimum of 10 mm from the breast surface for all examples, following the results of Petersson et al.^39^

In total, 21 simulations were run with cup A and 156 simulations with cup B. We simulated breast compression for the mediolateral (ML) mammographic view for all examples.

### 3.4 Simulation of Breast Deformation during Mammographic Compression

**Fig. 4:**
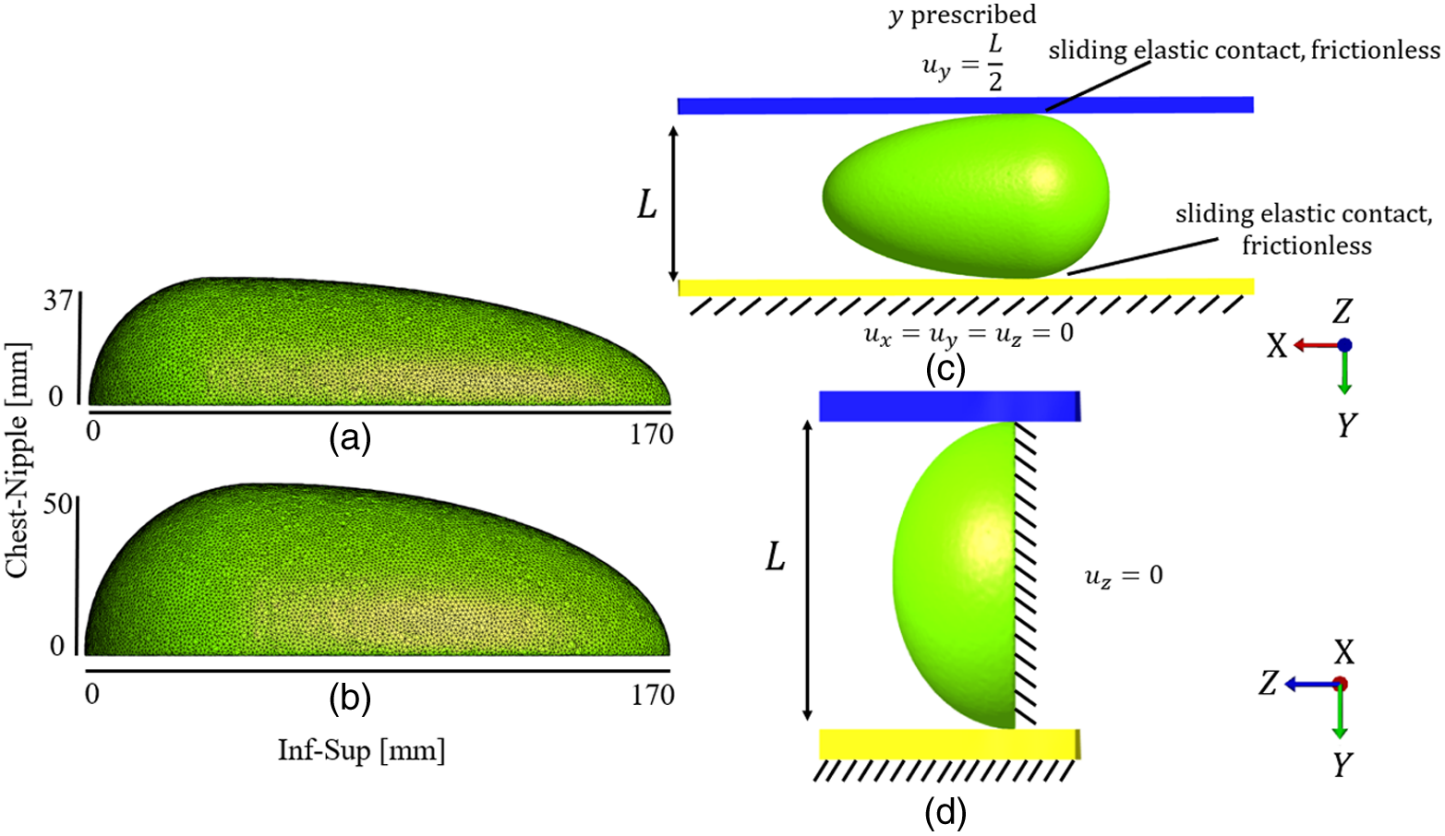
(a) The cup A (dimensions 170×75×37  mm) breast in the inferior-superior direction and chest-nipple direction (coronal-sagittal plane). (b) The cup B (dimensions 170×100×50  mm) breast in the inferior-superior direction and chest-nipple direction (coronal-sagittal plane). (c) Boundary conditions for the FE model in the coronal-transversal plane. (d) Boundary conditions for the FE model in the sagittal-transversal plane. The top blue plate is the mobile compression plate, and the bottom yellow plate is the fixed breast support. The dashed lines mark restricted movement.

Breast compression was simulated by deforming the breast between two rigid bodies (compression plate and breast support). The breast support was fixed in all directions, and the compression plate had a prescribed negative displacement in the y-direction (transversal plane), corresponding to compressing the breast to 50% of its original thickness. The contact between the breast and both rigid bodies was defined as “sliding elastic” and frictionless. The nodes comprising the chest wall were fixed in the z-direction (chest-nipple direction). One node in contact with the compression plate was restricted in all directions to prevent rigid body movement. The boundary conditions were the same for both cups A and B [Figs. 4(c) and 4(d)].

### 3.5 Validation of Simulated Mechanical Imaging Acquisition

To enable comparison between simulated and clinical data, a simulated sensor map with 1×1  cm sensor elements in a grid pattern was created from the FE results. The sensor was located on the compression plate [blue plate in Figs. 3(c), 3(d), 4(c), and 4(d)]. For comparative purposes with clinical data, the y-stress was used as the primary output for stress from all FE simulations.

The RMPA values were defined as described in Sec. 2. The RMPA for each simulation was defined as the RMPA of the tile that contained the center of the lesion and was calculated for each lesion location and lesion stiffness.

The average stress at the breast surface in the direction of compression and the average stress at the lesion location on the surface were calculated for comparison with clinical results.^40^ The average stress at the lesion location was defined as the area on the breast surface projected from the lesion location. This was repeated for each lesion location and lesion stiffness. We compared the average stress at the breast surface and the average stress at the lesion location and assessed the statistical significance of the difference using Wilcoxon signed-rank, as the data were not normally distributed.

Our simulation results were compared with the range of average stress over the breast surface and lesion location as reported in previous clinical MI investigations.^40^^,^^41^

## 4 Results

### 4.1 Simulated Breast Deformation during Mammographic Compression

**Fig. 5:**
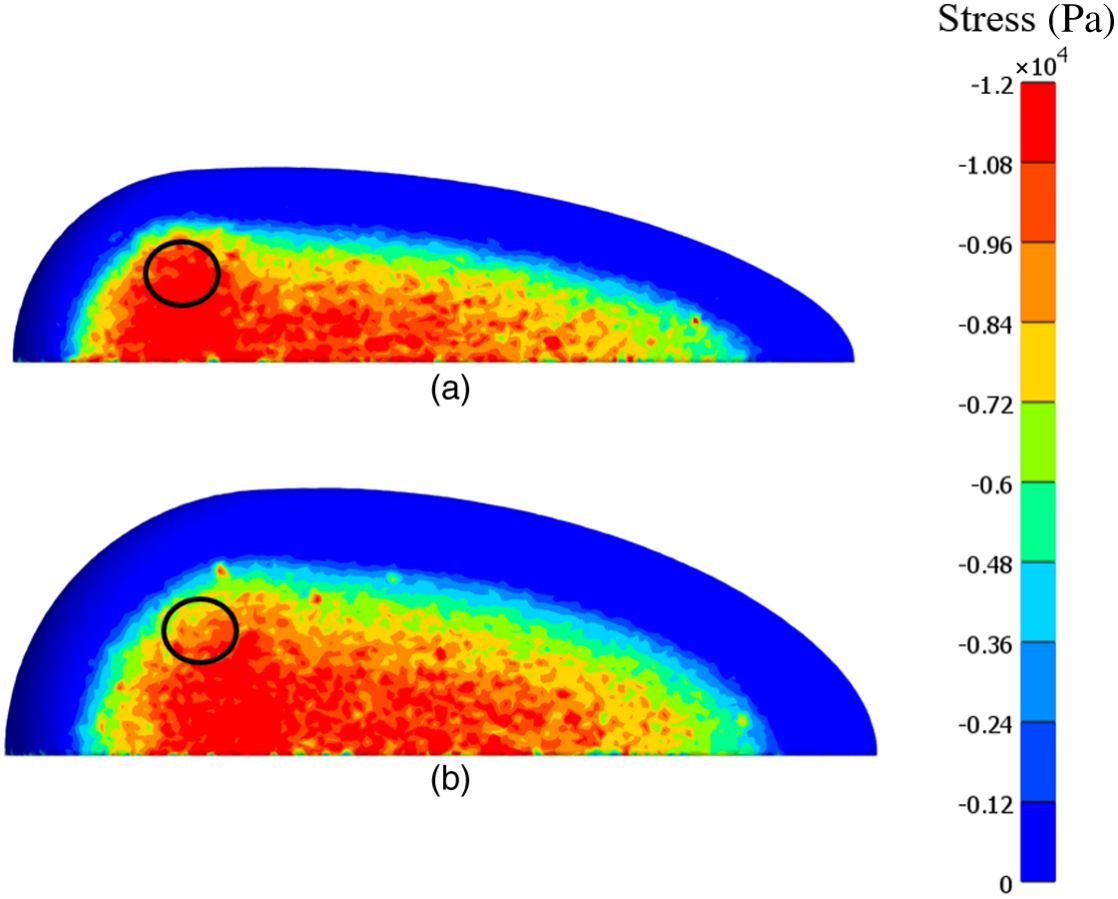
(a) Stress distribution of cup A at full compression. The lesion location is marked with a circle, and the lesion is 50 times stiffer than the adipose tissue. (b) Stress distribution of cup B at full compression. The lesion location is marked with a circle, and the lesion is 50 times stiffer than the adipose tissue.

Two examples of the stress distribution over the compressed breast surface for cups A and B can be seen in Figs. 5(a) and 5(b), respectively.

The average stress at the lesion location for cup A was 10.05±3.13  kPa (range 5.93 to 14.50 kPa), 11.05±3.80  kPa (range 6.30 to 15.90 kPa), and 11.43±4.10  kPa (range 6.23 to 16.60 kPa) for lesion stiffnesses of 15, 30, and 50 times stiffer than the adipose tissue, respectively. The average stress at the breast surface was 6.20±0.02  kPa (range 6.17 to 6.24 kPa), 6.23±0.04  kPa (range 6.20 to 6.31 kPa), and 6.24±0.05  kPa (range 6.16 to 6.24 kPa) for a lesion 15, 30, and 50 stiffer than the adipose tissue, respectively (Table 1). The standard deviation was calculated over the set of average stress values for all different lesion locations, given the same lesion stiffness. The Wilcoxon signed-rank test showed that the average stress at the breast surface (column 2 in Table 1) was significantly lower (p-values in column 4, Table 1) than the average stress at the lesion location (column 3 in Table 1).

**Table 1:** Average stress at the breast surface and at the lesion location for lesion stiffnesses that were 15, 30, and 50 times stiffer than adipose tissue for the cup A model, and the p-value when comparing the average stress at the breast surface with the average stress at the lesion location.

Lesion stiffness	Average stress at breast surface (kPa)	Average stress at lesion location (kPa)	Statistical significance (p<0.05)
15	6.20±0.02	10.05±3.13	0.031
30	6.23±0.04	11.05±3.80	0.016
50	6.24±0.05	11.43±4.10	0.016

The average stress at the lesion location for cup B was 7.81±1.00  kPa (range 5.88 to 10.30 kPa), 7.95±1.00  kPa (range 6.00 to 10.45 kPa), and 8.04±1.00  kPa (range 6.18 to 10.60 kPa) for lesion stiffnesses 15, 30, and 50 times stiffer than adipose tissue, respectively. The corresponding average stress at the breast surface was 6.43±0.04  kPa (range 6.40 to 6.52 kPa), 6.45±0.05  kPa (range 6.31 to 6.53 kPa), and 6.46±0.07  kPa (range 6.16 to 6.54 kPa) (Table 2). The Wilcoxon signed-rank test showed that the average stress at the breast surface (column 2 in Table 2) was significantly lower (p-values in column 4, Table 2) than the average stress at the lesion location (column 3 in Table 2).

**Table 2:** Average stress at the breast surface and at the lesion location for lesion stiffnesses that were 15, 30, and 50 times stiffer than adipose tissue for the cup B model, and the p-value when comparing the average stress at the breast surface with the average stress at the lesion location.

Lesion stiffness	Average stress at breast surface (kPa)	Average stress at lesion location (kPa)	Statistical significance (p<0.05)
15	6.43±0.04	7.81±1.00	$1\times 10^{-9}$
30	6.45±0.05	7.95±1.00	$7\times 10^{-10}$
50	6.46±0.07	8.04±1.00	$2\times 10^{-4}$

**Fig. 6:**
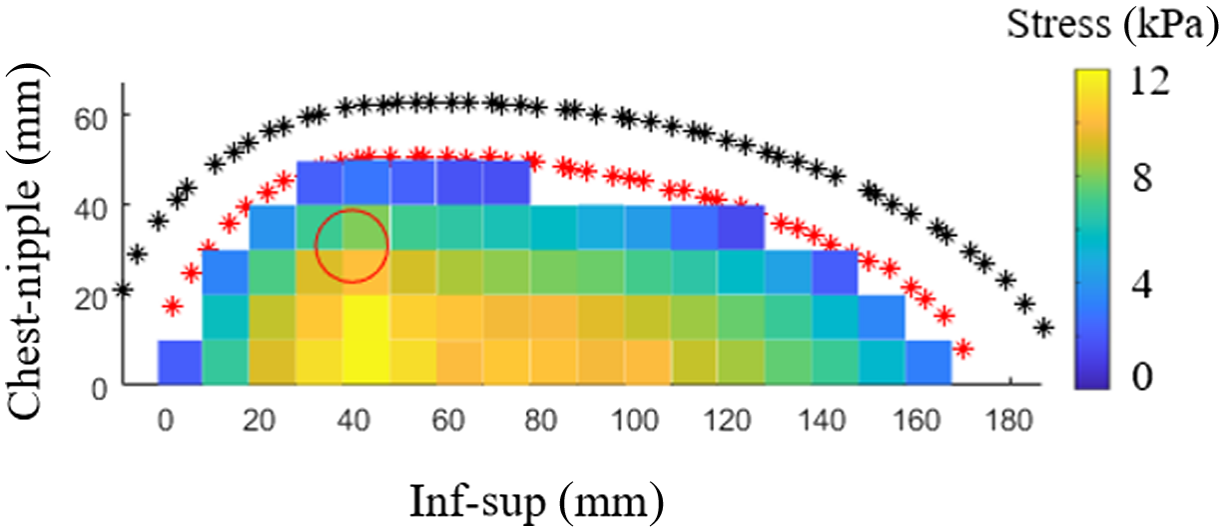
The simulated sensor response from the FE results (example based on the cup B model) from making a 1×1  cm grid of the stress over the breast surface given in Fig. 5(b). The red outline marks the breast surface in contact with the compression plate, and the black outline marks the thickest part of the breast at full compression. The red circle marks the lesion location and size.

The simulated sensor response using the FE results to resemble the MI sensor is presented in Fig. 6.

### 4.2 Analysis of Stress and RMPA as a Function of Lesion Location

**Fig. 7:**
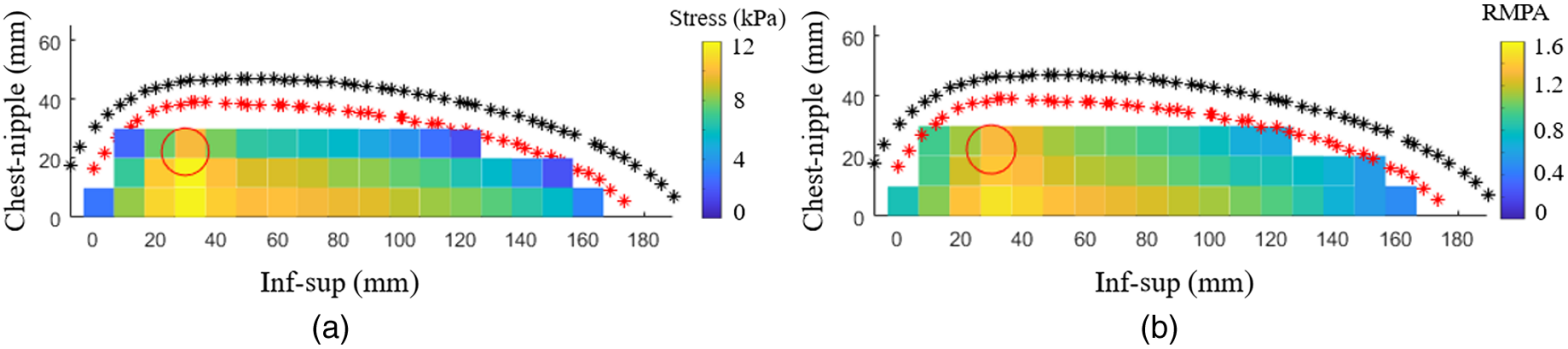
(a) The simulated sensor response from the FE results (cup A), and the (b) corresponding RMPA of the stress map. The red outline marks the breast surface in contact with the compression plate, and the black outline marks the thickest part of the breast at full compression. The red circle marks the lesion location and size. The lesion is 50 times stiffer than the surrounding tissue.

The RMPA values for the cups A and B breasts ranged from 0.80 to 1.46 and 0.98 to 1.34, respectively. The RMPA for most examples increased with increasing lesion stiffness. An example of the RMPA values over the breast using the simulated sensor response can be seen in Figs. 7(a) and 7(b).

**Fig. 8:**
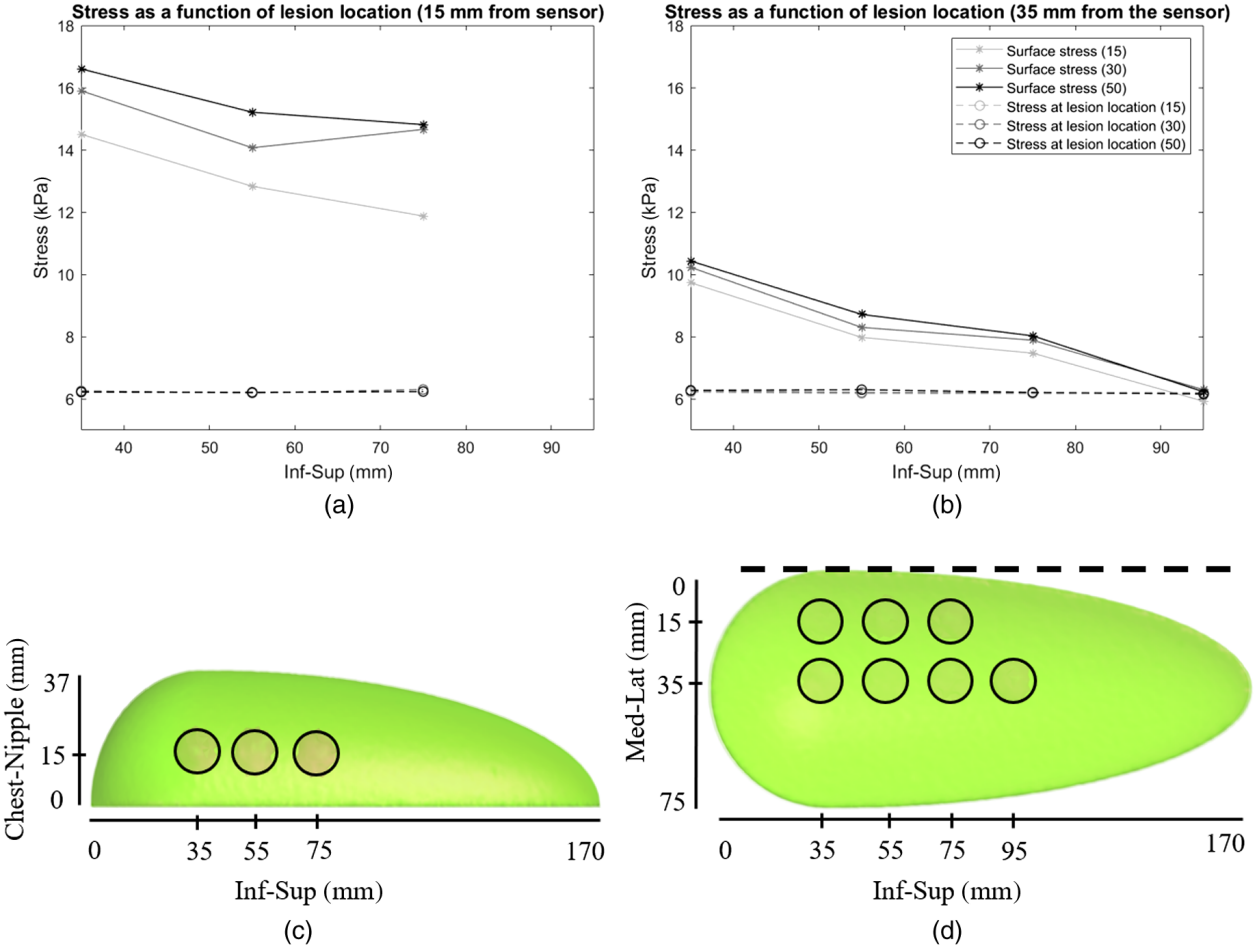
Stress as a function of lesion location. (a) Stress as a function of lesion location for lesions 15 mm from the sensor (d) and 15 mm from the chest wall (c) for lesion stiffnesses 15, 30, and 50 times stiffer than the surrounding adipose tissue (cup A breast). The full lines indicate the stress at the lesion location (each data point is the average stress at a specific lesion location for a specific lesion stiffness, marked with an asterisk), and the dashed lines indicate the stress over the entire breast surface (each data point is the average stress over the breast surface for a specific lesion location for a specific lesion stiffness, marked with a circle). (b) Stress as a function of lesion location for lesions 35 mm from the sensor (d) and 15 mm from the chest wall (c), for lesions that were 15, 30, and 50 times stiffer than the surrounding adipose tissue (cup A breast). (c) Lesion locations in the inferior-superior direction and chest-nipple direction (cup A model). The circle marks the lesion location. (d) Lesion locations in the inferior-superior direction and medial-lateral direction (cup A model). The circle marks the lesion location. The dotted line marks the sensor location.

The stress as a function of lesion location for cup A showed that the stress over the lesion location increased with increasing lesion stiffness [Figs. 8(a) and 8(b)]. Moreover, the stress at the lesion location was higher when the lesion was in closer proximity to the compression plate than when it was further away. The average stress at the breast surface varied very little with increasing lesion stiffness.

**Fig. 9:**
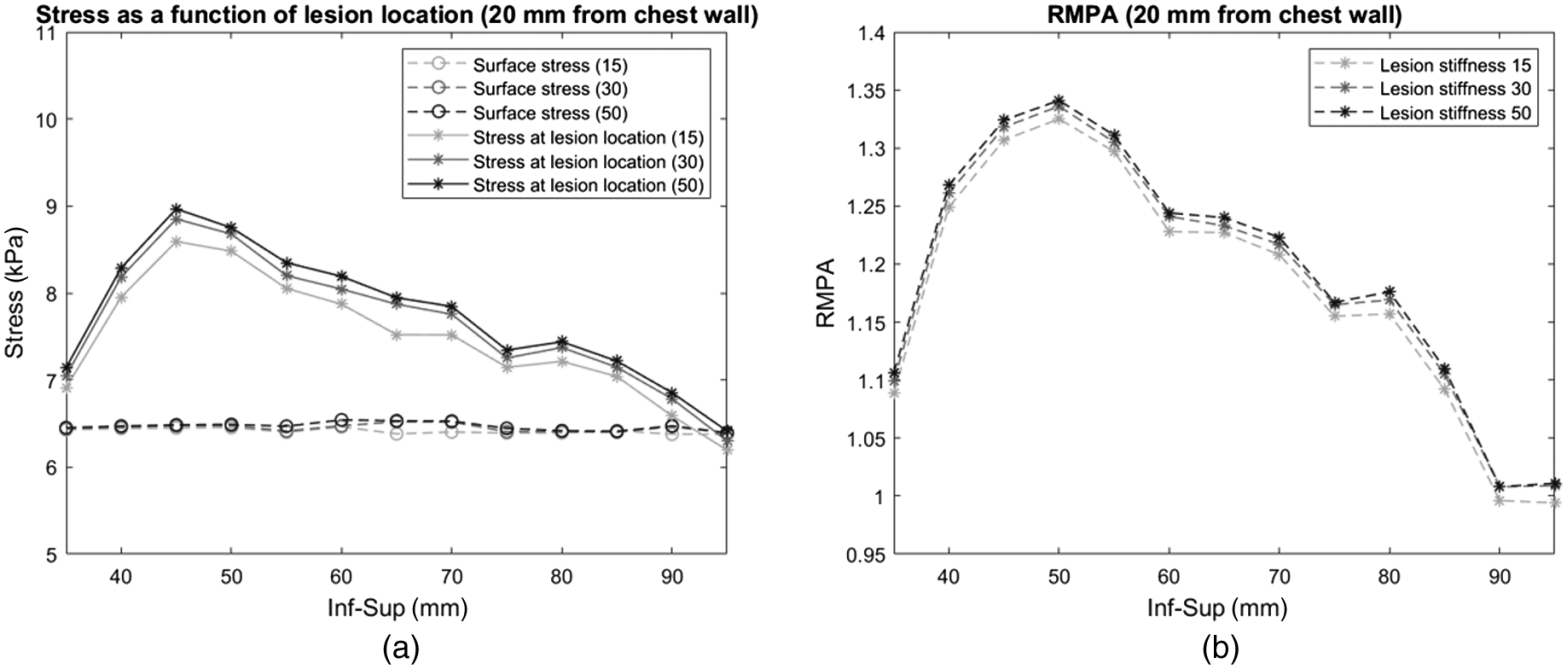
(a) Stress as a function of lesion location for lesions in the midbreast plane parallel to the sensor [Fig. 3(d)] and 20 mm from the chest wall [Fig. 3(c)], for lesions that were 15, 30, and 50 times stiffer than the surrounding adipose tissue (cup B breast). The full lines indicate the stress at the lesion location (each data point is the average stress at a specific lesion location for a specific lesion stiffness, marked with an asterisk), and the dashed lines indicate the stress over the entire breast surface (each data point is the average stress over the breast surface for a specific lesion location for a specific lesion stiffness, marked with a circle). (b) The corresponding RMPA values for lesions that were 15, 30, and 50 times stiffer, respectively. Each data point is the average stress at the lesion location divided by the average stress over the breast surface.

The stress as a function of lesion location for cup B showed much of the same behavior as cup A. The stress at the lesion location increased with increasing lesion stiffness while the average stress at the breast surface varied little [Fig. 9(a)]. The corresponding RMPA as a function of lesion location showed similar behavior as the stress over the lesion location [Fig. 9(b)].

### 4.3 Comparison of Simulated and Clinical Mechanical Imaging Data

As stated in Sec. 3.5, we compare the average stress over the lesion location and breast surface for our simulations with previously reported clinical MI data.^40^^,^^41^ The average stress values over the lesion varied 7.81 to 11.43 kPa (range 5.93 to 16.6 kPa), for all lesion stiffnesses and breast sizes combined. The average stress over the breast surface varied 6.20 to 6.46 kPa (range 6.16 to 6.54 kPa) for all lesion stiffnesses and breast sizes combined. This is in comparison with reported clinical values, with the average stress over the lesion location being 6.8±5.3  kPa (range 1.0 to 22.5 kPa).^40^ The average stress over the breast surface was 3.4±1.6  kPa (range 1.5 to 7.1 kPa) and 5.6±2.0  kPa (range 2.1 to 11.5 kPa).^40^^,^^41^

## 5 Discussion

The aim of this study was to model MI acquisition by simulating mammographic compression of a computer model of the breast anatomy and a lesion. Using the results from the FE models, we were able to calculate the average stress at the lesion location and average stress over the breast surface, and we simulated the sensor response and the RMPA maps.

In this work, we used a finite element model for a focused investigation of the stress map over the breast surface, as motivated by clinical MI investigation.^40^^,^^41^ Other reported works have instead used a finite element model to approximate the shape of the deformed breast to use in x-ray simulations,^14^^,^^18^^,^^20^^,^^25^^,^^29^^,^^33^ or for tracking the deformation of the breast or its internal structures.^15^^–^^17^

The average stress at the lesion location was roughly 20% to 50% higher than the average stress over the total breast surface. The average stress at the lesion location increased with increasing lesion stiffness for the majority of the lesion locations, as expected. For most simulations, the average stress at the lesion location decreased with increasing distance from the compression plate to the sensor. The average stress at the lesion location was higher for cup A than cup B. This was also expected as the lesion volume is relatively smaller in the larger breast and the positions for all inserted lesions are located further away from the compression plate, resulting in lower stress values.

The average stress over the breast surface did not increase as much with increasing lesion stiffness for either simulated breast sizes, as expected from clinical data. There was little variation of average surface stress overall for all simulated examples despite the increasing stiffness of the lesion. An explanation could be that the increase in the area of local high stress at the lesion location is not sufficiently large to affect the average stress on the breast surface significantly. This could in turn be due to the assumed linear elastic behavior of the breast tissue.

The simulated average stress over the breast surface was 6.20 to 6.46 kPa (see Tables 1 and 2), corresponded well to the reported clinical values by Förnvik et al.^41^ and Dustler et al.,^40^ that were 3.4±1.6  kPa (range 1.5 to 7.1 kPa) and 5.6±2.0  kPa (range 2.1 to 11.5 kPa), respectively. Our data was within, but on the higher end of, both reported clinical ranges. The obtained higher stress values may be caused by the choice of the mechanical properties of the adipose tissue in our simulations.

Förnvik et al.^41^ also reported the average mean stress at the lesion location to be 6.8±5.3  kPa (range 1.0 to 22.5 kPa). In our results, the stress at the lesion location was on average 7.81 to 11.43 kPa (see Tables 1 and 2). Here, our results are within the range of clinically reported values albeit once more on the higher end.

In a study by Dustler et al.,^27^ the reported clinical values for average stress over the breast surface in the ML-oblique (MLO) view when compressed with a rigid compression plate was 0.5 kPa (confidence interval 0.2 to 0.6). This is a substantially lower stress value than our simulation results. Moreover, stress values as high as 14.2 kPa for MLO at full compression have been reported by de Groot et al.,^42^^,^^43^ which is higher than our simulated results. Thus, there is a large variation in the ranges of values reported in the literature, which is most likely due to the use of different methods for defining the breast surface and measuring the pressure on it. It could also be due to a variation in the compression force applied by the radiographers.

We also note that the ranges of stress on the breast in previously mentioned studies^27^^,^^40^^,^^41^ have a larger variation than our simulation results. Our models assume only adipose tissue with homogenous mechanical properties throughout the breast, which could explain the small range of variation in our simulated results compared with the clinically reported ranges. Introducing different tissue composition, with variation in the mechanical properties, would probably lead to a wider range of values, as seen in the clinical data. We will address this in future studies to enable mimicking tissue compositions of specific patients.

Another potential way of validating the models would be to study the force over the breast. The reaction force in the direction of compression for cup A was 22 N and for cup B was 39 N. Clinical data from Dustler et al.^27^ reported that the force on the breast for MLO view with a rigid plate at full compression was in the range 3 to 34 N with breast thickness varying between 45 and 109 mm. The thickness of the cup A model at full compression was 35 mm, and for cup B it was 50 mm. In another study by Dustler et al.,^40^ the force with a rigid plate at full compression was 48±19  N (range 15 to 110 N). Our values are within the range of the reported forces. However, the force from the compression plate and the force over the entire breast are the same in our simulated examples because of the way in which the breast and compression paddle are modeled. This is in contrast with what was reported by Dustler et al.,^27^ in that the force of the compression plate on the breast (measured through the internal system of the x-ray imaging device) was higher than the derived force from the pressure measurements, i.e., the force on the breast surface.

The calculated RMPA values in this study ranged between 0.80 and 1.46. Clinically reported RMPA values by Dustler et al.^11^ for biopsy-proven benign findings was 1.3, which was found not to be statistically different from other benign findings with an RMPA of 1.0. The minimum threshold of RMPA for malignant cases was 0.6, but when excluding ductal carcinoma *in situ* and non-Hodgkin’s lymphoma, the threshold was 1.4. For some malignant cases, the RMPA value has been recorded as high as 6.1.

For the cup A model, the majority of the cases exceeded the RMPA threshold of 1.0 for nonbiopsied benign findings, and a few examples exceeded the minimum RMPA threshold for both the biopsy-proven benign and malignant findings. The cup B model had a maximum RMPA value of 1.34, which reaches the minimum threshold for benign biopsied findings, although most of the cases were below this threshold as they were in the range of 1.10 to 1.25. These findings indicate that the difference in stiffness between malignant and benign tissue is larger than what has been done in our study.

For the majority of the simulations, the RMPA increased with increasing lesion stiffness. However, despite there being an increase in RMPA, the increase was not significant. For each increase in lesion stiffness, the RMPA increased by roughly 1% to 2% for both models. For the cup B model, all simulations followed the behavior of an increased RMPA with increasing lesion stiffness except for a few outliers where the RMPA was slightly lower despite having higher lesion stiffness. There is also a pattern for the majority of the simulations in that the highest RMPA values are when the lesion is located in the thickest part of the breast.

There are several factors affecting our RMPA values. First is the impact that the distance to the compression plate with the sensor has on the stress at the lesion location and in turn the RMPA. If we define our breast surface as the surface in contact with the breast support [yellow plate in Figs. 4(c) and 4(d)] instead of the compression plate [blue plate in Figs. 4(c) and 4(d)] and study the lesion locations furthest away from the compression plate, we see that the RMPA increases when this change is implemented. This resulted in an increase in RMPA of roughly 7%.

Another factor is the resolution of the simulated stress map, which could explain the behavior of the outliers mentioned previously. If a lesion is split between four sensor elements/tiles, the stress profile of the lesion will be split over these four tiles, making the behavior less pronounced. Moreover, if the lesion center is split between several different tiles, it is hard to reliably determine which tile represents the lesion center. To address this issue, we analyzed the center location of all lesions in cups A and B. A total of 57% of the lesions for cup A had their center located within the tile, >2  mm away from the edge of the tile. For cup B, only 15% of the lesions had their center >2  mm from the edge of the tile.

One limitation of the current study is the assumption of a linear elastic material description for the tissues in the breast. Fung showed that most tissues in the breast exhibit hyperelastic behavior, in which the relationship between stress and strain is nonlinear.^44^ However, adipose tissue has been shown to have more of a linear response instead of hyperelastic.^10^ To get an approximation of the stiffness of the lesion tissue, ratios reported in the literature^10^^,^^37^ between lesion and adipose tissue were used. Hyperelasticity could improve the modeling and result in higher values of RMPA, most likely closer to those reported clinically.

We assumed mechanical properties of the adipose tissue based on data by Gefen and Dilmoney where the Elastic modulus was in the ranges of 0.5 to 25 kPa.^36^ This is similar to the ranges published by other researchers, who have reported Elastic modulus values of 18±7  kPa at a precompression of 5% and 20±8  kPa for 20% precompression for adipose tissue,^10^ and 10.4±7.9  kPa at 10% strain, respectively.^37^ Lower values of Elastic modulus have also been reported at around 3 kPa at low strain.^45^ The reason for the variability in the measured stress could be due to the different strain rates as well as the difference in the experimental setup.

For the mechanical properties of intraductal carcinoma, Krouskop et al.^10^ reported an Elastic modulus of 106±32  kPa at 5% precompression and 558±180  kPa at 20% precompression with a loading frequency of 0.1 Hz, and Wellman^37^ reported 385±127  kPa at 10% strain. Others reported much lower values at 23±3  kPa at low strain,^45^ showing a wide range of measured stiffnesses. As for the ratio between adipose and intraductal carcinoma, at 20% precompression Krouskop et al.^10^ showed it to be as much as 30 times stiffer than adipose tissue. Wellman^37^ showed that at 10% strain the carcinoma could be 40 times stiffer than adipose tissue and >70 times stiffer at 15% strain. It is also worth pointing out that, in the studies mentioned above, the tissue samples were measured *ex vivo*, which most likely results in different mechanical properties of the tissue than if they were measured *in vivo*. Similar values have however been reported in vivo using ultrasound (supersonic shear imaging) for adipose tissue of ca 3 to 5 kPa, around 45 kPa for the parenchyma, and malignant lesions above 100 kPa and as high as 180 kPa.^46^

Another limitation was that we only included one tissue type for normal breast tissue in our models, i.e., adipose tissue. Also, we did not take the complex connections of various tissue types into account, such as Cooper’s ligaments, for example, which have been shown to be important for the overall structural integrity of the breast.^47^

The model presented in this study is a first step toward an MI acquisition framework. Our future work will include describing the tissues’ mechanical behavior as hyperelastic and including multiple breast tissue types and compositions for more realistic mechanical behavior.

The model could be used to investigate the efficiency of MI, for example, on tumor depth, which is difficult to investigate clinically. Through this, we could aid in the optimization of MI’s clinical use and increase the specificity of breast cancer screening. The model could also be used to improve the accuracy of simulated breast images for VCTs.

## 6 Conclusion

We have simulated mechanical imaging applying the finite element method on computer models of breast anatomy and lesions. Overall, our results corresponded to the available clinical data regarding the average stress over the breast surface, average stress at the lesion location, and RMPA. With our mechanical imaging model, we obtained the average stress over the breast and the lesion location, which corresponded well to clinical measurements. The proposed model can be used in VCTs for the evaluation and optimization of mechanical imaging screening strategies.
